# Evaluation of biological pathways involved in chemotherapy response in breast cancer

**DOI:** 10.1186/bcr2088

**Published:** 2008-04-29

**Authors:** Attila Tordai, Jing Wang, Fabrice Andre, Cornelia Liedtke, Kai Yan, Christos Sotiriou, Gabriel N Hortobagyi, W Fraser Symmans, Lajos Pusztai

**Affiliations:** 1 Department of Breast Medical Oncology, The University of Texas M. D. Anderson Cancer Center, PO Box 301439, Houston, TX 77230-1439, USA; 2 Department of Molecular Diagnostics, National Medical Center, Szabolcs utca 33-35, 1135 Budapest, Hungary; 3 Department of Bioinformatics and Computation Biology, The University of Texas M. D. Anderson Cancer Center, PO Box 301439, Houston, TX 77230-1439, USA; 4 Unit UPRES03535, Institut Gustave Roussy, 39 Rue Camille Desmoulins, 94805 Villejuif, France; 5 Jules Bordet Institut, 121 Boulevard de Waterloolaan, Brussels 1000, Belgium

## Abstract

**Introduction:**

Our goal was to examine the association between biological pathways and response to chemotherapy in estrogen receptor-positive (ER^+^) and ER-negative (ER^-^) breast tumors separately.

**Methods:**

Gene set enrichment analysis including 852 predefined gene sets was applied to gene expression data from 51 ER^- ^and 82 ER^+ ^breast tumors that were all treated with a preoperative paclitaxel, 5-fluoruracil, doxorubicin, and cyclophosphamide chemotherapy.

**Results:**

Twenty-seven (53%) ER^- ^and 7 (9%) ER^+ ^patients had pathologic complete response (pCR) to therapy. Among the ER^- ^tumors, a proliferation gene signature (false discovery rate [FDR] q = 0.1), the genomic grade index (FDR q = 0.044), and the E2F3 pathway signature (FDR q = 0.22, *P *= 0.07) were enriched in the pCR group. Among the ER^+ ^tumors, the proliferation signature (FDR q = 0.001) and the genomic grade index (FDR q = 0.015) were also significantly enriched in cases with pCR. Ki67 expression, as single gene marker of proliferation, did not provide the same information as the entire proliferation signature. An ER-associated gene set (FDR q = 0.03) and a mutant p53 gene signature (FDR q = 0.0019) were enriched in ER^+ ^tumors with residual cancer.

**Conclusion:**

Proliferation- and genomic grade-related gene signatures are associated with chemotherapy sensitivity in both ER^- ^and ER^+ ^breast tumors. Genes involved in the E2F3 pathway are associated with chemotherapy sensitivity among ER^- ^tumors. The mutant p53 signature and expression of ER-related genes were associated with lower sensitivity to chemotherapy in ER^+ ^breast tumors only.

## Introduction

Drug resistance is caused by multiple mechanisms that operate simultaneously in tumors. A large number of biological functions, including transmembrane trafficking, DNA repair, stress response, proliferation, and apoptosis, may affect the sensitivity of a cell to chemotherapy. Other, yet-to-be-identified mechanisms may also play a role. Preoperative chemotherapy provides an attractive clinical setting to study mechanisms of drug resistance in patients.

Chemotherapy before surgery is used in the treatment of newly diagnosed, stage II-III breast tumors because it frequently reduces tumor size and improves surgical outcome [1]. Among patients who receive preoperative chemotherapy, up to 25% to 30% (depending on the type of treatment) experience complete eradication of the invasive cancer in the breast and regional lymph nodes after completion of 3 to 6 months of chemotherapy [2]. This favorable response is called pathologic complete response (pCR) and it indicates an extremely chemotherapy-sensitive tumor and also heralds excellent long-term cancer-free survival [3]. We previously conducted a pharmacogenomic study that included 133 patients with newly diagnosed breast cancer who received preoperative chemotherapy with paclitaxel followed by 5-fluorouracil, doxorubicin, and cyclophosphamide. All patients underwent a one-time, pretreatment, fine-needle biopsy of the cancer for gene expression analysis. The goal of the study was to discover gene-expression-based predictors of pCR. Our previous analysis focused on discovering the best possible multigene predictor without considering the function of any of the genes [4]. The goal of the present analysis is to examine an association between known biological pathways and response to chemotherapy.

Lists of genes (that is, gene sets) that represent various biological pathways were assembled from the literature. We used gene set enrichment analysis (GSEA) to examine the correlation between these *a priori*-defined gene sets and chemotherapy response [5]. Clinical experience as well as molecular analysis of breast tumors indicate that estrogen receptor-positive (ER^+^) and ER-negative (ER^-^) tumors are two different types of neoplastic disease of the breast [6,7]. It is plausible that different molecular mechanisms may determine response or resistance to chemotherapy in these two types of breast tumor. Therefore, we performed our analysis separately for ER^+ ^and ER^- ^tumors.

## Materials and methods

### Patients

This study included 51 ER^- ^and 82 ER^+ ^tumors from patients with newly diagnosed stage I-III breast cancer. Each patient had a fine-needle aspiration of the cancer before starting chemotherapy. These needle aspiration samples contain approximately 80% neoplastic cells and few or no stromal cells or normal breast epithelium [8]. All patients were treated with 6 months of preoperative chemotherapy with paclitaxel followed by 5-fluorouracil, doxorubicin, and cyclophosphamide. Patients underwent surgery after completion of chemotherapy, and the resection specimens were examined by a pathologist to measure residual cancer. For the purpose of our analysis, tumor response was dichotomized as pCR, defined as no residual invasive cancer in the breast and lymph nodes, or as residual disease (RD), which included patients with any degree of invasive cancer that survived preoperative chemotherapy. The reason for this dichotomization was that pCR is a strong surrogate for long-term cancer-free survival and therefore a marker of long-term benefit from therapy [2,3]. It remains unknown to what extent patients who achieve less than pCR benefit from chemotherapy in terms of improved survival. This categorization of pathologic response allowed us to compare biological pathways between tumors with extreme chemotherapy sensitivity (pCR) and the rest (RD). There were not enough cases with tumor progression during treatment in our study to form a third group including extreme chemotherapy-resistant tumors.

ER status was determined from routine pathological assessment by immunohistochemistry. Following standard clinical practice, the cutoff for ER positivity was greater than or equal to 10% positive tumor cells. This study was approved by the institutional review boards of the M. D. Anderson Cancer Center (Houston, TX, USA), and all patients signed an informed consent form for voluntary participation. Clinical characteristics of the patients are presented in Table 1.

**Table 1:** Patient characteristics

Characteristics	ER-negative group (n = 51)	ER-positive group (n = 82)
Age, years		
Median	51	51
Range	29–75	28–79
T stage		
T0	0	1 (1%)
T1	8 (16%)	4 (5%)
T2	24 (47%)	46 (56%)
T3/4	19 (37%)	31 (38%)
Histological grade		
Grade 1	0	2 (2%)
Grade 2	6 (12%)	45 (55%)
Grade 3	43 (84%)	31 (38%)
Unknown	2 (4%)	4 (5%)
Lymph node status		
Positive	38 (75%)	55 (67%)
Negative	13 (25%)	27 (33%)
HER2 overexpressed or amplified		
Yes	18 (35%)	15 (18%)
No	32 (63%)	67 (82%)
Unknown	1 (2%)	0
Pathologic complete response		
Yes	27 (53%)	7 (9%)
No	24 (47%)	75 (91%)

ER, estrogen receptor.

### Gene expression analysis

Gene expression profiling was performed by using Affymetrix U133A Gene Chips (Affymetrix, Santa Clara, CA, USA) following standard operating procedures as described previously [4]. We normalized the gene expression data using dChip V1.3 software [9] to a single reference array. The normalized gene expression values were transformed to a log_10 _scale for further analysis. The complete microarray data are available at the M. D. Anderson Cancer Center bioinformatics website [10]. To identify differentially expressed genes between cases with pCR and RD, we performed the unequal variance *t *test on each probe set. Because of the multiple comparisons, many low *P *values are expected by chance alone. Under the null hypothesis that no genes provide useful information, the distribution of *P *values should be uniform. If, on the other hand, some genes do provide useful information about predicting response, we would expect an overabundance of small *P *values (above what chance might produce). We can capture this situation by modeling the distribution of the *P *values as a beta-uniform mixture (BUM). This analysis was used to estimate false discovery rates (FDRs) that accompany particular *P *values derived from the *t *test [11]. All analysis was performed using the R package (version 2.3.1).

### Gene set enrichment analysis

GSEA was applied to assess the association between pCR, RD, and 852 distinct *a priori*-defined gene sets. The goal of the GSEA is to determine whether members of a particular gene set (that is, a list of 15 to 500 probe sets that correspond to genes that define a biological pathway) tend to occur toward the top or the bottom of a rank-ordered gene list including all gene expression measurements [5]. We ranked all probe sets based on their correlation with pCR. Three groups of gene sets were tested: The first included 319 distinct gene sets corresponding to probes associated with 295 different cryptogenic bands on 24 chromosomes, and the second included 522 different gene sets corresponding to genes involved in various metabolic and signaling pathways. A detailed description of these gene sets and how they were assembled was presented by Subramanian and colleagues [5]. The third group contained 11 gene sets of various oncogenic or drug-resistance-related pathways. These included 5 distinct oncogenic pathways that were defined as genes overexpressed in normal human mammary epithelial cells transfected with Myc, Ras, E2F3, β-catenin, and Src oncogenes, respectively, and were described by Bild and colleagues [12]. We also included a mutated p53-associated gene set that was defined as genes overexpressed in p53 mutant compared to p53 normal breast cancers [13]. It has been suggested that this gene expression signature can distinguish tumors with wild-type and mutant p53 and it may outperform direct p53 gene sequencing as a predictor of prognosis and therapeutic response. We also assessed one ER-associated gene set that contained genes that were most highly coexpressed with the ER gene in human breast cancer microarray data developed by Symmans and colleagues [14]. This gene set did not include the ER gene itself. We also examined the genomic grade index (GGI) that represents genes that are differentially expressed between low-grade and high-grade human breast tumors and that were identified by Sotiriou and colleagues [15]. One prognostic signature that was derived by comparing gene expression profiles of tumors that recurred with those that did not was also tested. This prognostic signature was first reported by Wang and colleagues [16]. A proliferation signature set reported by Whitfield and colleagues [17] that includes genes involved in cell proliferation was also examined. Finally, we also assessed an ATP-binding cassette transporter (ABC) gene set that included genes involved in drug transport and was previously shown by Szakacs and colleagues [18] to predict chemotherapy response in cell lines.

Gene annotations were based on UniGene Build 185, which was used to match the genes in each of the above publications to probe sets on Affymetrix U133A Gene Chips. The gene sets are listed in Table 2, and a complete list of all probe sets that comprise each of the 852 sets is provided in Supplementary Table 1.

**Table 2:** Gene sets used in this analysis

Functional pathway	Number of probe sets (corresponding number of known genes)	Reference
Cytogenetic sets, n = 319 sets	15–500 (variable)	Subramanian, *et al*. [5]
Functional sets, n = 522 sets	15–500 (variable)	Subramanian, *et al*. [5]
Oncogenic pathways, 5 sets		Bild, *et al*. [12]
Myc	164 (139)	
Ras	228 (176)	
E2F3	173 (147)	
β-catenin	54 (42)	
Src	46 (44)	
Genomic grade index, 1 set	242 (183)	Sotiriou, *et al*. [15]
76-gene prognostic signature, 1 set	76 (76)	Wang, *et al*. [16]
Proliferation signature, 1 set	74 (44)	Whitfield, *et al*. [17]
ABC transporter gene set, 1 set	61 (47)	Szakacs, *et al*. [18]
Mutant p53 signature, 1 set	25 (21)	Miller, *et al*. [13]
Estrogen receptor-associated gene set, 1 set	200 (187)	Symmans, *et al*. [14]

Gene set enrichment score was calculated as reported previously [5]. This score is a measure of the degree to which a gene set is over-represented at the extremes of the entire ranked gene list. Significance was assessed by permuting class labels (that is, response category) and calculating enrichment scores for the permuted data sets that yielded a null distribution. Nominal *P *value for a score was derived from comparison with this null distribution. To adjust for multiple hypothesis testing, the FDR q value was calculated for each gene set. The q value could be considered as an FDR-adjusted *P *value. However, unlike *P *values, which express the probability of a false-positive result for a single test, the q value gives an estimate of the proportion of false positives for a set of results [19]. Gene sets with an FDR q value of less than or equal to 0.25 were considered to be of interest, which indicates that the result is likely to be valid three out of four times and represents a previously proposed cutoff in the literature [5]. GSEA was performed using the R package of GSEA (version 1.0) provided by the Broad Institute of the Massachusetts Institute of Technology (Cambridge, MA, USA).

## Results

### Differentially expressed genes

**Figure 1:**
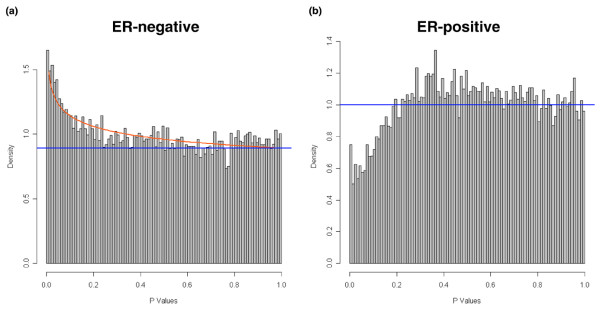
Distribution of *P *values computed from the unequal variance *t *test in patients with estrogen receptor (ER)-negative and ER-positive tumors. **(a) **Gene expressions were compared between ER-negative tumors that had pathologic complete response and those that had a lesser response to preoperative chemotherapy. The resulting *P *values for all comparisons were modeled as beta-uniform mixture. The straight line indicates the contribution of the uniform component, and the curved line is the fitted beta-distribution from the observed values. Deviation above the straight line indicates *P *values that may represent true discovery. **(b) **Distribution of *P *values in patients with ER-positive tumors.

Twenty-seven out of 51 patients (53%) had pCR among the ER^- ^tumors, and 7 out of 82 (9%) among the ER^+ ^tumors. The much lower response rate in ER^+ ^tumors is consistent with previous reports in the clinical literature [1-4]. First, we examined whether we could identify differentially expressed genes between cases with pCR and RD by means of the unequal variance *t *test. We performed this analysis separately for ER^- ^and ER^+ ^tumors. Figure 1 shows results of the BUM analysis of the *P *values from the *t *test. In ER^- ^tumors, the FDR associated with the lowest *P *value (*P *≤ 0.00087) was 40%. In ER^+ ^tumors, the FDR was close to 100% for all observed *P *values. The scarcity of low *P *values in the ER^+ ^group is due to the unbalanced sample size (that is, few informative cases, 7 pCR only) and suggests an underpowered analysis that violates *t *test assumptions.

These observations indicate that, in these two data sets, the *t *test cannot reliably identify differentially expressed genes. However, these results do not necessarily indicate that there are no real transcriptional differences between cases with pCR compared with RD when ER^+ ^and ER^- ^tumors are analyzed separately. It is possible that no individual gene meets the threshold for statistical significance after correcting for multiple hypothesis testing because the transcriptional differences are modest relative to the technical noise and biological variability that are present in the data. Analysis at the single gene level may also miss small but coordinated expression differences in a larger number of genes that could belong to important biological pathways. In some situations, small coordinated change in the expression of many genes that belong to a particular metabolic pathway can have robust functional consequences [20]. Such subtle gene expression differences would not be identified easily by pairwise comparisons using *t*-statistics. Different analytical tools, including GSEA, were developed to test for potentially relevant but small-scale transcriptional differences in predefined sets of genes.

### Gene set enrichment analysis to identify pathways associated with complete response to preoperative chemotherapy

**Figure 2:**
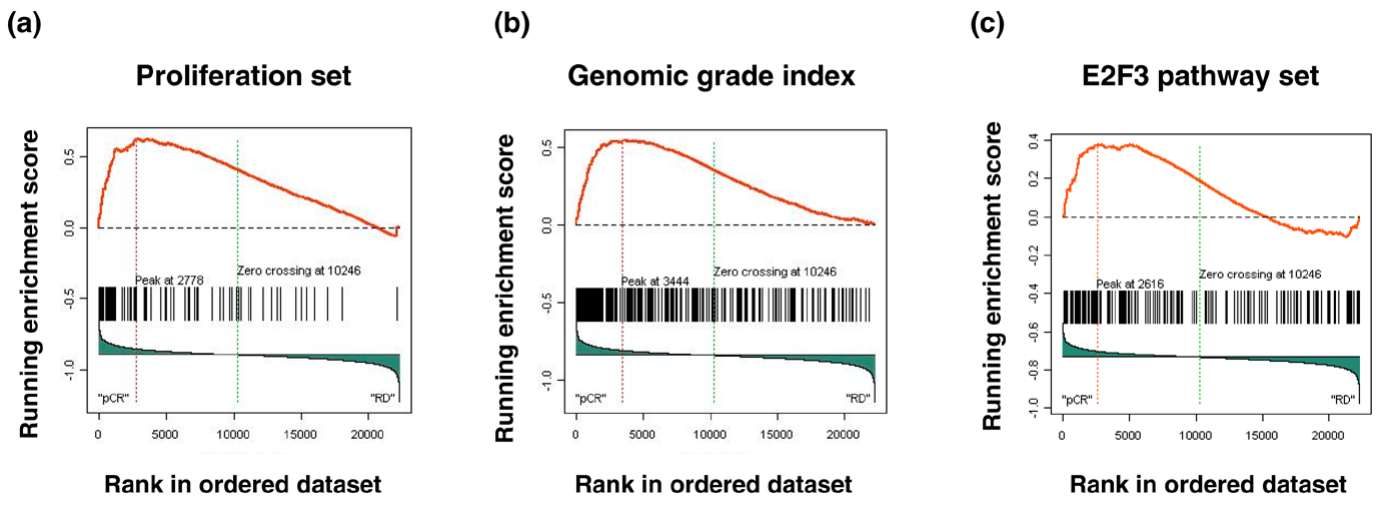
Gene set enrichment results for estrogen receptor-negative breast tumors. Running enrichment scores (RESs) and the location of each probe set within the complete rank-ordered gene list for each gene set. The dotted line on the left indicates the position of the maximum RES, and the dotted line on the right indicates the zero position of the ranking metric score. **(a) **Proliferation set (probe set n = 74). **(b) **Genomic grade index (probe set n = 242). **(c) **E2F3 pathway (probe set n = 173). Heat maps corresponding to these plots are provided in Supplementary Figure 1. pCR, pathologic complete response; RD, residual disease.

We applied GSEA to the 51 ER^- ^tumors. Only 3 gene sets out of the 853 were enriched with an FDR q value of less than or equal to 0.25. These gene sets included (a) the proliferation set (FDR q = 0.1, *P *= 0.05) (Figure 2a and Supplementary Figure 1a), (b) the GGI set (FDR q = 0.04, *P *= 0.08) (Figure 2b and Supplementary Figure 1b), and (c) the E2F3 pathway gene set (FDR q = 0.2, *P *= 0.07) (Figure 2c and Supplementary Figure 1c). All of these were enriched in the group with pCR, whereas no gene set was enriched in the group with residual cancer.

**Figure 3:**
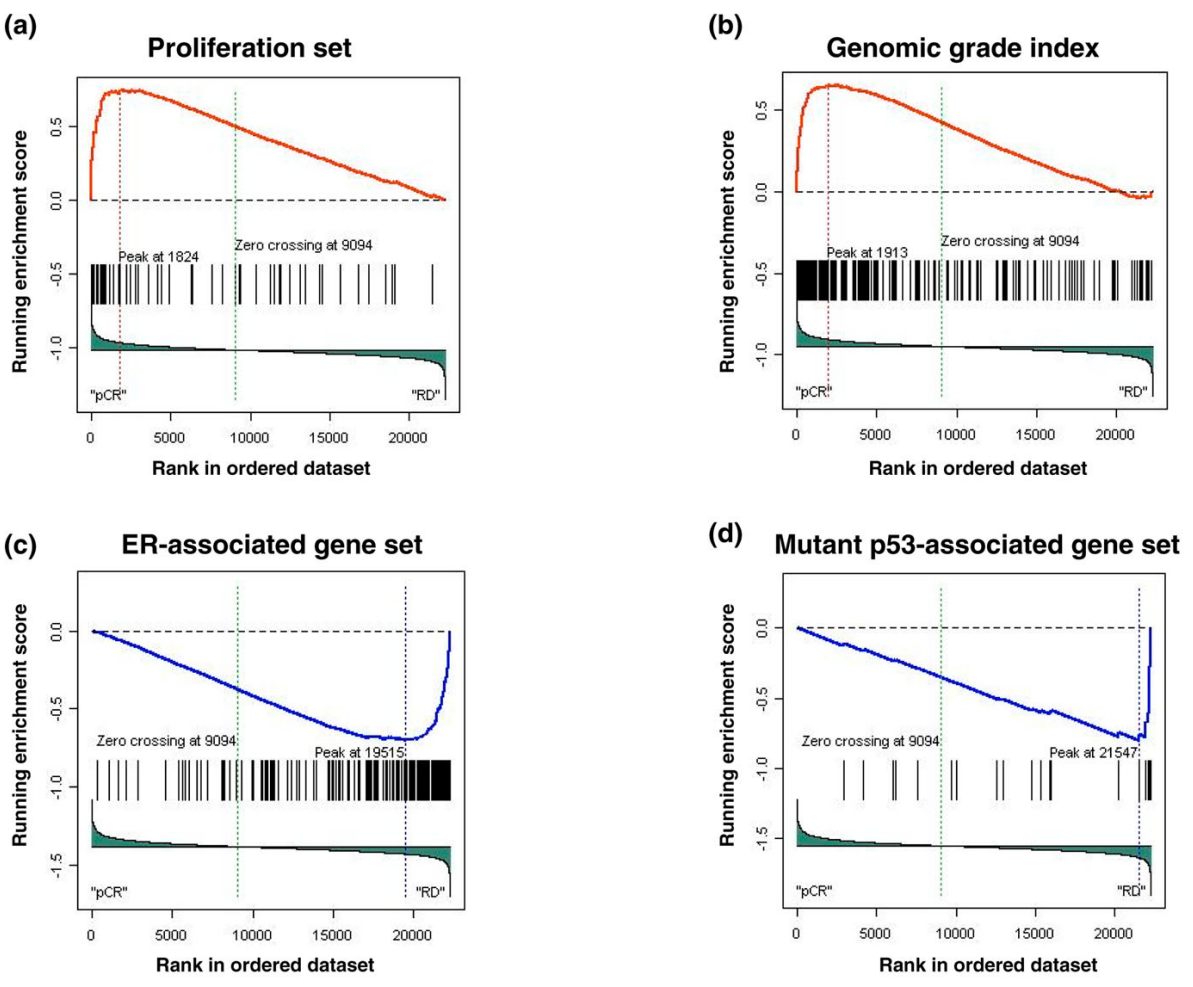
Gene set enrichment results for estrogen receptor (ER)-positive breast tumors. Results are presented as in Figure 2. **(a) **Proliferation set. **(b) **Genomic grade index. **(c) **ER-associated genes (probe set n = 201). **(d) **Mutant p53 gene signature (probe set n = 25). Heat maps corresponding to these plots are provided in Supplementary Figure 2. pCR, pathologic complete response; RD, residual disease.

We performed the same analysis on the 82 ER^+ ^cases. Two gene sets were enriched in the group with pCR: the proliferation set (FDR q = 0.001, *P *= 0.002) (Figure 3a and Supplementary Figure 2a) and the GGI set (FDR q = 0.015, *P *= 0.01) (Figure 3b and Supplementary Figure 2b). In the group with residual cancer, two other gene sets showed enrichment: the ER-associated gene list (FDR q = 0.03, *P *= 0.04) (Figure 3c and Supplementary Figure 2c) and the mutant p53 gene signature (FDR q = 0.0019, *P *= 0.07) (Figure 3d and Supplementary Figure 2d). The complete list of probes and genes included in the five enriched gene sets is presented in Supplementary Table 2.

These results indicate that higher expression of proliferation-related genes characterized tumors with pCR among both ER^- ^and ER^+ ^tumors. There are several single gene markers of proliferative activity; the one that is used most commonly in the clinic is Ki67 (which was also included in the proliferation gene set). We therefore examined whether measuring Ki67 (MIK67) mRNA expression alone is sufficient to separate cases with pCR from those with RD after chemotherapy. Ki67 expression is measured by two distinct Affymetrix probe sets: '212021_s_at' and '212023_s_at'. In ER^- ^tumors, neither of these probe sets was significantly differentially expressed according to pathologic response to chemotherapy (unequal variance *t *test, *P *= 0.97 and 0.92, respectively). In ER^+ ^tumors, one of the two probe sets ('212023_s_at') showed borderline significant overexpression in the pCR group (*P *= 0.06).

Also, since the ER-associated gene set was enriched in ER^+ ^breast tumors with RD after chemotherapy, we examined whether quantitative assessment of the ER (ESR1) mRNA alone could provide the same information [21]. There was no statistically significant difference in ER mRNA expression levels (probe set '205225_at') between cases with complete response and those with residual cancer after chemotherapy (unequal variance *t *test, *P *= 0.09) among the ER^+ ^tumors.

## Discussion

In the present study, we examined whether we could find individual genes or gene sets that are significantly associated with extreme chemotherapy sensitivity in ER^+ ^and ER^- ^breast tumors, respectively. These two major types of breast tumor differ in the expression of thousands of genes [6,7,22]. They also have substantially different sensitivity to cytotoxic treatment; ER^+ ^tumors are generally less sensitive to chemotherapy than ER^- ^tumors [2]. Therefore, when these tumors are analyzed together, sensitivity markers tend to identify ER^- ^tumors and are often dominated by genes that reflect the ER status of the tumor [4]. To identify markers of response that are independent of ER status, we analyzed these two groups of breast tumors separately. To our surprise, the commonly used approach which performs genewise comparison between responders and nonresponders failed to identify any genes that could be declared differentially expressed with statistical confidence. The estimated FDR was greater than 40% among the top differentially expressed genes in ER^- ^tumors and the FDR was even higher among ER^+ ^tumors. These findings are in contrast with the results that can be obtained when the entire patient cohort is analyzed together. When we searched for differentially expressed genes including both ER^- ^and ER^+ ^cases, we could identify over 400 genes with an FDR of less than or equal to 1% [4].

We next examined whether coordinated but relatively small-scale differences in the expression of sets of genes that belong to functional pathways are associated with response. Such small-scale differences at the individual gene level may not be readily identified by *t*-statistics but GSEA may be able to detect these. Two gene sets emerged as strongly enriched in tumors with pCR to chemotherapy in both ER^+ ^and ER^- ^tumors. These included 44 genes (corresponding to 74 probe sets) involved in cell proliferation and 183 genes (corresponding to 242 probe sets) that distinguish histologically high-grade tumors from low-grade tumors. These observations are consistent with the literature that suggests that highly proliferative tumors are more sensitive to cytotoxic treatment in general [23]. However, there is no consensus on how to best measure proliferative activity [24]. To underscore the power of gene set analysis, we noted that the proliferation signature as a whole was significantly over-represented in highly chemotherapy-sensitive tumors. However, a commonly used proliferation marker, Ki67, which was included in the signature, showed no significant overexpression when tested alone. It is also well documented in the clinical literature that high histological grade is associated with better response to preoperative chemotherapy [25]. It was reassuring to observe that the same association holds up for the GGI too.

We also made three novel observations. Our results indicate that the expression of genes involved in the E2F3 pathway may be associated with a high degree of chemotherapy sensitivity in ER^- ^tumors. Given that the E2F3 family of transcription factors plays a critical role in regulating cell cycle progression, this association is not surprising [26]. Nevertheless, no previous reports linked E2F3 activity to chemotherapy response. It is also intriguing that no association between E2F3 pathway and pCR was seen in ER^+ ^tumors. We also observed higher expression of mutant p53-associated genes in relatively chemotherapy-resistant ER^+ ^breast tumors. A similar association was not seen among ER^- ^tumors, which suggests that p53 dysregulation may have different consequences on chemotherapy sensitivity depending on the hormone receptor status of the tumor. This may partly explain the conflicting results about the role of p53 mutation in chemotherapy response in the literature. Some studies suggested that functional p53 defects predict for increased sensitivity to anthracycline chemotherapy [27]. Others reported that p53 mutations are associated with resistance to anthracyclines [28,29]. It has also been shown that breast cancer cell lines exhibit different transcriptional response to chemotherapy *in vitro *depending on their hormone receptor status and molecular class. For example, the expression of p21, a p53-regulated protein, was highly induced in ER^+ ^cells but only weakly induced in ER^- ^breast cancer cell lines in response to anthracycline exposure [30]. This suggests that p53-mediated apoptosis may be more important in ER^+ ^(luminal) than in ER^- ^(basal-like) cells. Future biomarker studies will need to consider the possibility that the predictive value of a biomarker may depend on the molecular subtype of the tumor [31].

We also observed that those ER^+ ^tumors that had low expression of ER-associated genes were more sensitive to chemotherapy. This was independent of the actual level of ER expression and indicates that some ER^+ ^breast tumors do not posses the full transcriptional signature of ER activity. These tumors showed increased chemotherapy sensitivity.

Our study has limitations. All patients received combination chemotherapy that represents the current standard of care for this patient population. This makes our observations more relevant for clinical practice but at the same time limits our ability to decipher drug-specific response pathways. This could have biased our results toward detecting 'generic' drug sensitivity pathways such as proliferation. The gene sets that we tested were assembled from the published literature and often contain overlapping genes represented in multiple gene sets. Our current knowledge of biology is incomplete and does not allow the precise definition of all the genes that contribute to a given biological process or represent a unique molecular pathway. This may explain why the majority of the 852 gene sets that we examined, including numerous apoptosis and signaling pathways, did not show enrichment by chemotherapy response. It is also important to consider that GSEA is a method to demonstrate that the expression of a given gene set is over-represented in the top or bottom of particular gene lists ranked by correlation with clinical outcome. However, this method cannot be used to predict response in a new case. How to translate GSEA results into a prospective single-sample response predictor remains an unsolved bioinformatics challenge.

## Conclusion

We found that it is difficult to identify individual genes associated with chemotherapy response with statistical confidence when ER^- ^and ER^+ ^breast tumors are analyzed separately. In contrast, GSEA revealed several biological pathways that were associated with response. These included proliferation-related genes and the GGI that were enriched in tumors with high sensitivity to chemotherapy regardless of ER status. Genes included in the E2F3 pathway were also enriched in ER^- ^and highly chemotherapy-sensitive tumors. On the other hand, a mutant p53 gene expression signature and a set of highly ER-associated genes were enriched in ER^+ ^and chemotherapy-resistant tumors. These results suggest that proliferative activity confers increased sensitivity to chemotherapy in breast cancer in general, whereas other biological pathways such as p53 mutation and E2F3 activation may be more selective and influence chemotherapy sensitivity only in particular molecular subtypes of breast cancer.

## Abbreviations

BUM = beta-uniform mixture; ER = estrogen receptor; FDR = false discovery rate; GGI = genomic grade index; GSEA = gene set enrichment analysis; pCR = pathologic complete response; RD = residual disease.

## Competing interests

The authors declare that they have no competing interests.

## Authors' contributions

AT, FA, CL, and LP participated in developing the idea of the study, assembling the molecular and clinical data, performing the evaluation of results, preparing the figures, and drafting and finalizing the manuscript. JW participated in developing the idea of the study, assembling the molecular and clinical data, performing the evaluation of results, preparing the figures, performing the statistical analyses, and drafting and finalizing the manuscript. KY participated in performing the statistical analyses. WFS generated the gene expression data and helped to draft and revise the manuscript. CS and GNH helped to draft and revise the manuscript. All authors read and approved the final manuscript.
